# Comprehensive Analysis of the Influence of Expanded Vermiculite on the Foaming Process and Selected Properties of Composite Rigid Polyurethane Foams

**DOI:** 10.3390/polym14224967

**Published:** 2022-11-16

**Authors:** Mateusz Barczewski, Maria Kurańska, Kamila Sałasińska, Joanna Aniśko, Joanna Szulc, Izabela Szafraniak-Wiza, Aleksander Prociak, Krzysztof Polaczek, Katarzyna Uram, Karolina Surmacz, Adam Piasecki

**Affiliations:** 1 Institute of Materials Technology, Poznan University of Technology, Piotrowo 3, 61-138 Poznan, Poland; 2 Department of Chemistry and Technology of Polymers, Cracow University of Technology, Warszawska 24, 31-155 Cracow, Poland; 3 Faculty of Materials Science and Engineering, Warsaw University of Technology, Wołoska 141, 02-507 Warsaw, Poland; 4 Central Institute for Labour Protection—National Research Institute, Department of Chemical, Biological and Aerosol Hazards, 00-701 Warsaw, Poland; 5 Faculty of Chemical Technology and Engineering, Bydgoszcz University of Technology, Seminaryjna 3, 85-326 Bydgoszcz, Poland; 6 Institute of Materials Engineering, Faculty of Materials Engineering and Technical Physics, Poznan University of Technology, Piotrowo 3, 61-138 Poznan, Poland

**Keywords:** polyurethane foams, PUR, vermiculite, rigid foams, thermal insulation

## Abstract

This article presents the results of research on obtaining new polyurethane (PUR) foams modified with thermally expanded vermiculite. The filler was added in amount of 3 wt.% up to 15 wt.%. The additionally applied procedure of immersion the non-organic filler in H_2_O_2_ was performed to increase the exfoliation effect of thermally treated mineral and additional oxidation the surfaces. The effect of fillers on foaming process, cell structure, thermal insulation, apparent density, compressive strength, thermal properties, and flammability are assessed. The foaming process of PUR foams modified with vermiculite was comparable for all systems, regardless of the content of the filler. A slight increase in reactivity was observed, confirmed by a faster decrease in dielectric polarization for the system with modified vermiculite by H_2_O_2_. The modification of the reference system with the vermiculite increased the content of closed cells from 76% to 91% for the foams with the highest vermiculite content. Coefficient of thermal conductivity of reference foam and foams modified with vermiculite was in the range 24–26 mW/mK. The use of vermiculite up to 15 wt.% did not influence significantly on mechanical properties and flammability, which from an economic point of view is important because it is possible to reduce the cost of materials by introducing a cheap filler without deteriorating their properties.

## 1. Introduction

Rigid polyurethane (PUR) foams are mainly used as high-performance thermal insulation of buildings, refrigerators and transmission pipes [1]. Rigid PUR foams with apparent densities of about 30 to 200 kg/m^3^ withstand temperatures between −196 °C and 130 °C [2]. The thermal conductivity value of closed cells PUR foams ranges from 0.02 W/m∙K to 0.03 W/m∙K (influenced by the gas filling the foam cells [3]), which is a lower value compared to other commonly used thermal insulation materials, such as mineral wool (0.037–0.055 W/m∙K), cellulose (0.040–0.065 W/m∙K), expanded polystyrene (0.03–0.04 W/m∙K), and extruded polystyrene (0.034–0.044 W/m∙K) [1]. Currently, one of the main trends in research on rigid foams concerns the use of filler to modify foam properties, such as increasing mechanical strength or reducing thermal conductivity, flammability, or apparent density [1]. A promising filler that could find application in polyurethanes, including rigid and elastomeric solid materials and foams, is vermiculite (VMT) [4,5,6,7].

VMT is a commonly used layered silicate characterized by a single-layer structure in a 2:1 system, which consists of two layers of silicon oxygen tetrahedron sandwiched by layers of magnesium oxygen octahedron. The single layer is approximately 1 nm thick, while the interlayer spacing is usually around 1.4 nm [8]. As a result of the partial replacement of the silicon-oxygen tetrahedron sheet by aluminum, vermiculite has a negative charge and cations are found in the structure of the mineral, e.g., Ca^2+^, K^+^, or Mg^2+^, which maintain electrical balance in the interlayer [8,9]. This mineral is found at various latitudes and is mainly mined in South Africa [10], China [11], and Brazil [12]. Owing to its structure, this material offers a possibility of intensive volume growth after high-temperature heating, resulting in a product in the form of expanded vermiculite. Thermal expansion occurs perpendicularly to VMT sheets, and the product obtained after a thermal modification has a concertina-like, highly porous structure [8,13]. Thanks to the separation of sheets of VMT, including expanded VMT, this material is widely used in construction, as acoustic and thermal insulation, agriculture, and as a filler for polymeric materials [7,8,13,14,15]. The use of plate-shaped fillers allows for an increase in the barrier properties of polymers modified with them; this effect can be used both to increase the effectiveness of flame-retardant systems [5,16] and to reduce oxygen diffusion into closed cells of PUR foams, slowing down insulation aging [4]. To obtain the monosheets of the filler from vermiculite, it is necessary to break its packet and complex structure. This process may occur in thermal treatment, leading to the formation of expanded vermiculite, or exfoliation by organofunctionalization [8,14]. Most studies relate to the implementation of VMT organofunctionalization, which results from the possibility of giving new functional features and obtaining a controlled structure of the filler. Expanded vermiculite after mechanical processing (grinding and sieving) is characterized by a much lower price and the possibility of easy process implementation in industrial conditions. It should be mentioned that compared with expanded VMT, VMT nanosheets with larger specific surface areas and more reactive sites provide opportunities for exfoliated VMT to serve also as a nanocomposite material for nanofluidic channels and intelligent responses [8].

Among the published works on the modification of PUR with different varieties of vermiculite, most of the research has been aimed at improving the dispersion of the filler, increasing the mechanical and thermal properties of the final materials, as well as improving the barrier effects. In the studies of Zhang et al. [7], it was shown that a deliberate modification of VMT by cation exchange with octadecyl trimethyl ammonium bromide allowed increasing the ability of OVMT to disperse better in polyurethane, as well as to create of additional physical cross-links in the polycarbonate polyol structure that are constituent elements of PU soft-segments. As a result, materials with significantly increased (by more than 50%) tensile strength were obtained. In turn, Park et al. [4] modified the filler based on cation exchange with long-chain quaternary ammonium, allowing for increasing the filler dispersion in methylene diphenyl isocyanate (MDI). In the case of porous materials, the final properties of foams depend not only on the type of filler, dispersion, and polymer-filler interface interactions but also on the modification of the cell structure. Umasankar Patro and co-workers studied the effect of a nanometric exfoliated vermiculite addition on the properties of MDI-based rigid foams [6]. Investigations have shown that with an addition of 8 pphp of VMT, the target cell area was reduced by approximately 50%, with a simultaneous increase in the share of closed cells. This translated into a reduction in the thermal conductivity of composite foams compared to unmodified PUR foams and improved mechanical properties.

The present study assesses the effect of a micrometric filler in the form of expanded vermiculite, as a low-cost filler, on the thermal and mechanical properties and the flammability of rigid PUR foams. Moreover, research was undertaken to verify the validity of the application of an additional treatment consisting of the immersion of the filler in a concentrated hydrogen peroxide solution [17]. This process aimed to increase the exfoliation of the inorganic filler’s structure and the filler surface’s reactivity with the isocyanate component. The possibility of implementing a simple modification procedure and its impact on the thermoset matrix of PUR foams were analyzed.

## 2. Experimental

### 2.1. Materials

Polyether polyol based on sorbitol Rokopol RF-551, having a hydroxyl value of 400–440 mgKOH/g, a water content of 0.10 wt.%, a viscosity of 3000–5000 mPa∙s, and a functionality of 4.5, was supplied by PCC Rokita S.A. (Brzeg Dolny, Poland). Polycat 9 produced by Evonik Industries AG (Essen, Germany), was used as a catalyst. Niax silicone L-6633 supplied by Momentive Performance Materials Inc. (Waterford, NY, USA) was used as a stabilizer of the foam structure.

Polymeric 4,4′-diphenylmethane diisocyanate (PMDI) with a free isocyanate groups content of 31 wt.% was supplied by Minova Ekochem S.A. (Siemianowice Śląskie, Poland). Water was used as a chemical blowing agent, which in reaction with isocyanate generates carbon dioxide. LANXESS (Cologne, Germany) supplied a flame retardant, triethyl phosphate (TEP).

Thermally expanded vermiculite with a particle size of up to 1.6 mm was provided by Perlit Polska (Puńców, Poland). According to the producer’s data, the thermal treatment was carried out at a temperature of 1260 °C, and the chemical composition of the inorganic filler was as follows: 38.0–49.0% SiO_2_, 20–23.5% MgO, 12–17.5% Al_2_O_3_, 0.3–5.4% Fe_2_O_3_, 5.2–7.9% K_2_O, 0–1.2% FeO, 0.7–1.5% CaO, 0–0.8% Na_2_O, 0–1.5% TiO_2_, 0–0.5 Cr_2_O_3_, 0.1–0.3% MnO, 0–0.6% CI, 0–0.6% CO_2_, 0–0.2% S.

### 2.2. Filler Preparation

The use of thermal expansion usually allows for the process of water release and exfoliation of the vermiculite structure [8,13]. The additionally applied procedure of immersion of the non-organic filler in H_2_O_2_ was to boost the exfoliation effect of thermally treated mineral and additionally oxidize the surface, increasing the amount of isocyanate-reactive hydroxyl groups. The process was carried out within 24 h, which, according to the literature data, allows for obtaining an effective exfoliation effect of unmodified vermiculite [17]. Then, VMT was dried at the temperature of 80 °C for 48 h, and the remaining water was evaporated. The untreated thermally expanded vermiculite filler is marked with W, while the filler treated with hydrogen peroxide is designated as WO in the further part of the present study.

### 2.3. Preparation of Rigid Polyurethane Foams

A reference rigid PUR foam and the products modified with W and WO were prepared by a single-step method. The polyol premix consisting of a polyol, catalyst, surfactant, blowing agent, and vermiculite was mixed for 30 s. Next, the polyol premix and isocyanate were mixed for 6 s and poured into an open mold (250 mm × 250 mm), where they expanded freely in the vertical direction. The mass of vermiculite was 3%, 6%, 9%, 12%, and 15% of the polyol mass. The isocyanate index was 1.1. The materials were conditioned for 24 h at room temperature before being cut and tested. The formulation used for the preparation of PUR foams is shown in Table 1.

### 2.4. Methods

The particle size of the W and WO fillers was assessed by a laser particle sizer Fritsch ANALYSETTE 22 apparatus (Idar-Oberstein, Germany) operated in the range of 0.08–2000 µm. The cumulative size distribution Q3(x) and adequate histogram dQ3(x) were considered during the analysis.

The parameters of the porous structure of the inorganic fillers subjected to different treatments, such as nitrogen adsorption isotherms at −196.15 °C and surface area, were determined using an accelerated surface area and porosimetry apparatus Micromeritics ASAP^®^ 2420 (Norcross, GA, USA) by Brunauer–Emmett–Teller (BET) method. All samples were degassed at 120 °C for 12 h in a vacuum chamber prior to measurements. The specific surface area was determined by the multipoint BET method using adsorption data under relative pressure (p/p0).

The crystallographic structure of the materials was analyzed by the X-ray diffraction (XRD) with Cu Ka radiation (l = 1.54 Å) Panalytical, Empyrean model (Almelo, The Netherlands). The conditions of the XRD measurements were as follows: voltage 45 kV, anode current 40 mA, 2 Theta range from 5° to 40°, time per step 60.214 s, step size 0.0165°.

The viscosities of the polyol premixes filled with various amounts of vermiculite were found using a rotational rheometer MCR 301 from Anton Paar (Graz, Austria) operated with a 25 mm parallel plates measuring system with a gap of 0.3 mm. All specimens were pre-sheared before testing for 1 min with a shear rate of 1 s^−1^ and a subsequent relaxation time of 2 min. The measurements were realized in the constant shear mode using 0.1, 1, and 10 s^−1^ shear rates at 30 °C. The presented dynamic viscosity results are mean values from the 300 s experiment.

The foaming process was analyzed using the foam qualification system FOAMAT from Format Messtechnik GmbH (Karlsruhe, Germany), which allows determining changes in characteristic parameters of PUR reaction mixture, such as the temperature and dielectric polarization, during the foaming process.

The apparent density was measured as the ratio of the mass and volume of the samples according to ISO 845. The content of closed cells in the samples was measured in accordance with ISO 4590. The cell structure was examined with the use of a scanning electron microscope Hitachi S-4700 (Tokyo, Japan). The anisotropy index was calculated as the ratio of the cell heights and widths.

The compressive strength at 10% deformation was analyzed in accordance with ISO 826. The compressive strength of the foams was measured using an AllroundLine model Z005 TH from Zwick Roell (Austria)instrument in two directions, parallel and perpendicular to the rise direction of the foams. The compressive force was applied at a speed of 2 mm/s, axially in a normal direction to a square surface. The heat conduction coefficient tests were carried out using foam samples with dimensions of 200 × 200 × 50 mm and a FOX 200 apparatus produced in accordance with the ISO 8301 standard at an average temperature of 10 °C. The temperature of the cold plate was 0 °C, and the temperature of the warm plate was 20 °C.

The additional analysis of filler dispersion in the polyurethane matrix was conducted using the scanning electron microscope MIRA3 from Tescan (Brno, Czech Republic). The measurements were performed with an accelerated voltage of 12 kV in backscattered electrons (BSE) and the secondary electron (SE) mode. The thin carbon coating (~20 nm) was deposited on samples using JEE 4B vacuum evaporator from Jeol (Tokio, Japonia).

The color of the PUR samples was evaluated according to the International Commission on Illumination (CIE) through L*a*b* coordinates [18]. In this system, L* is the color lightness (L* = 0 for black and L* = 100 for white), a* is the green(−)/red(+) axis, and b* is the blue(−)/yellow(+) axis. The color was determined by optical spectroscopy using a MiniScan MS/S-4000S spectrophotometer from HunterLab (Reston, VA, USA) and placed in a specially designed light trap chamber. The total color difference parameter ΔE* was calculated according to the following formulation [19]:

∆E* = [(∆L*)^2^ + (∆a*)^2^ + (∆b*)^2^]^0.5^ (1)

The thermal properties of PUR were examined by thermogravimetric analysis (TGA) with the temperature set between 25 °C and 900 °C at a heating rate of 10 °C·min^−1^ under nitrogen atmospheres using a TG 209 F1 apparatus from Netzsch (Germany). Samples having masses of 10 mg ± 0.1 mg were placed in Al_2_O_3_ pans. The initial decomposition temperature Ti was determined as the temperature at which the mass loss was 5%. Additionally, temperatures at 10, 25, and 50% mass loss were found.

The limiting oxygen index (LOI) was determined according to ISO 4589-2:2017. Burning behavior was evaluated with the use of a cone calorimeter from Fire Testing Technology (East Grinstead, UK). The samples (100 × 100 × 25 mm) were placed in aluminum foil and tested horizontally at an applied heat flux of 35 kW/m^2^, in conformity with the ISO 5660 standard. Spark ignition was used to ignite the pyrolysis products. Next, the residues were photographed using a digital camera EOS 400 D from Canon Inc. (Tokyo, Japan).

## 3. Results

### 3.1. Filler Characterization

**Figure 1:**
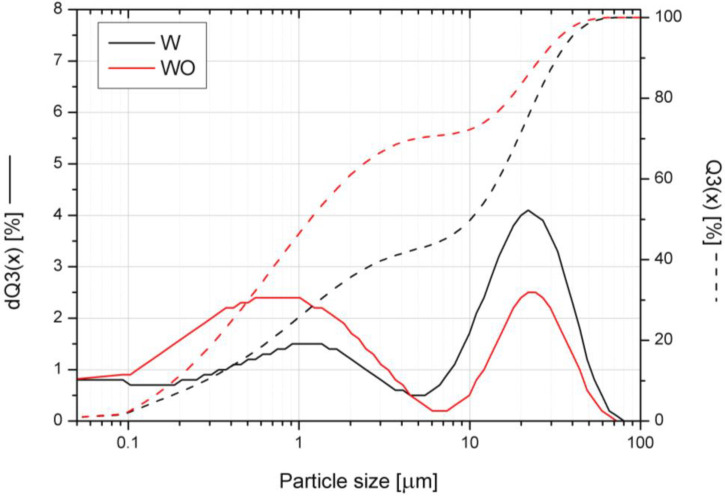
Particle size distributions for inorganic fillers.

The cumulative size distribution Q3(x) and adequate histograms dQ3(x) made for the inorganic fillers used in this study are presented in Figure 1. An analysis of the graphs shows that the ground and sieved expanded W is characterized by larger particle sizes compared to WO. Additional treatment using hydrogen peroxide allows increasing the content of particles with smaller sizes, which is probably due to the destruction of the concertina-shaped vermiculite structure and an additional exfoliation effect, confirmed by XRD evaluation. Both vermiculite grades (W and WO) exhibit two particle size distribution modes due to the fraction of finely divided filler sheets formed during the grinding process.

**Figure 2:**
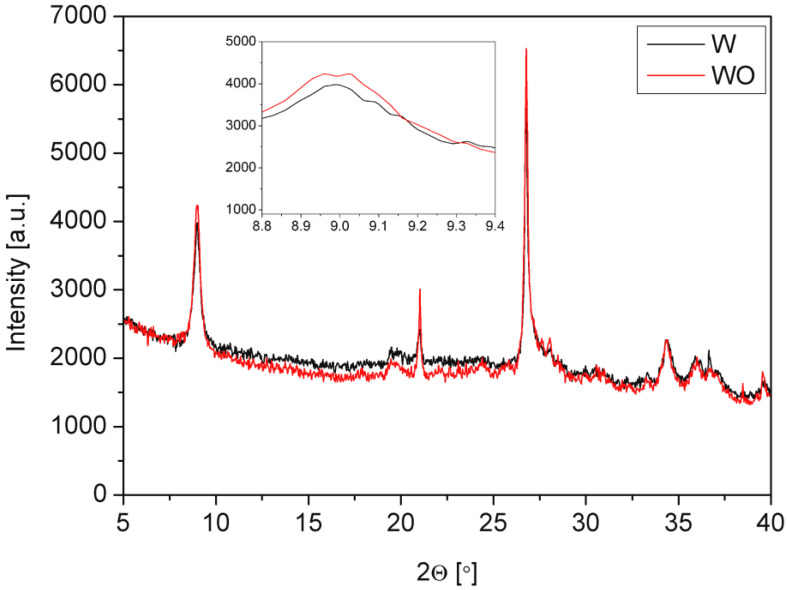
XRD patterns of thermally expanded vermiculite (W) and vermiculite additionally treated grade with H_2_O_2_ (WO).

In Figure 2, X-ray diffraction patterns of expanded vermiculite and vermiculite are also treated with hydrogen peroxide. The peaks at 2θ = 9°, 21.0°, 26.8°, and 34.3° in both vermiculites correspond to d-spacing of 9.8 Å, 4.2 Å, 3.3 Å, and 2.6 Å, respectively. The peak at 9° was shifted slightly to 8.96° after the treatment, which caused a d-spacing shift from 9.82° to 9.86°. This may be understood as an additional exfoliating effect of a chemical treatment [6]. The phenomenon may improve the dispersion of the filler in a polyol composition and the efficiency of modification of the final PUR foam.

**Figure 3:**
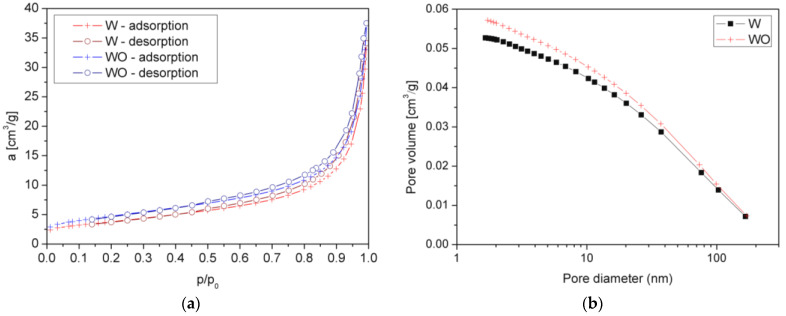
Nitrogen adsorption-desorption isotherms of thermally expanded vermiculite (W) and vermiculite additionally treated with H_2_O_2_ (WO).

BET surface area (S_BET_), t-Plot external surface area (S_EXT_), t-Plot micropore area (S_MIC_), desorption average pore width (4 V/A), single point desorption total pore volume of pores less than 12.1 nm diameter at p/p_0_ = 0.98325121 (V_P_), t-Plot micropore volume (V_MIC_) values are presented in Table 2. Figure 3a shows N_2_ adsorption-desorption isotherms of the filler before and after the H_2_O_2_ treatment. The course of the a(p/p_0_) curve is typical of expanded hydrous phyllosilicates [20,21]. The physicochemical properties found in our experiment are in good agreement with the literature [20]. S_BET_ increased after the two-step treatment (Table 2). Both fillers correspond to similar courses of the curves without other inflections and hysteresis loops, which may be related to significant changes in the modified material structure. Figure 3b presents a pore volume vs. pore diameter plot. It can be concluded that for both fillers, the pore diameter is below 40 nm. Therefore, the primary mechanism of adsorption results from mesopores adsorption. When p/p_0_ increases above 0.8, the adsorption increases significantly, suggesting the presence of micropores [22]. Both vermiculites can be described as type II in the Brauner classification. The measured adsorption in the whole considered range is higher for WO. The additional chemical treatment improved the physicochemical properties of the filler, including the specific surface area, which should cause an improved reactivity toward the chemically hardened polymer.

### 3.2. Rheological Properties

**Figure 4:**
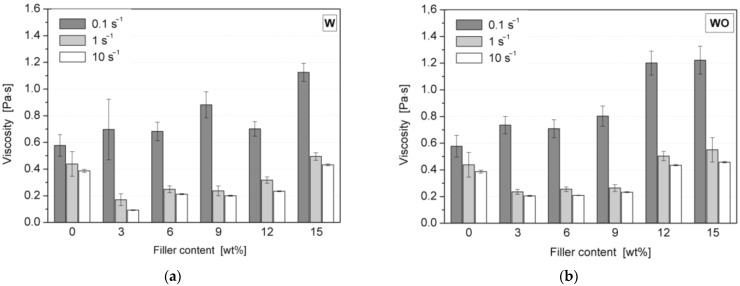
Rheological properties of polyol-W (**a**) and -WO (**b**) premixes with various filler contents measured with 0.1, 1, and 10 s^−1^ shear rates.

Figure 4 shows the results of viscosity measurements of polyol premixes containing various contents of expanded vermiculite without treatment (W) and after an additional peroxide treatment (WO).

It can be seen that an increasing content of the filler in polyol premix increases the dynamic viscosity of the composition, while at higher shear rates, the unfilled polyol shows lower viscosities. Usually, introducing a powder filler increases the viscosity [5,23]. It should be emphasized that the employed filler has an expanded form and was additionally fragmented with the use of a high-speed grinder and sieved. Therefore, in the considered case, the plate-shaped geometry of the filler and the measurement procedure, including the pre-shearing of the polyol, caused the observed changes in the average viscosity values. Consequently, at the lowest shear rate, the filler probably did not align itself with the flow direction, while the higher shear rates made the vermiculite sheets orient themselves, reducing the viscosity with respect to the unfilled composition. The compositions containing W exhibited greater spreads of the recorded mean viscosity values, which is understandable because of larger filler particles and a broader particle size distribution. It should be noted that the addition of vermiculite significantly increased the viscosity of the compositions only for a low shear rate. Therefore, the systems studied here may find spray-forming applications without affecting the processing conditions.

### 3.3. Foaming Process of PUR Systems Modified with Vermiculite

**Figure 5:**
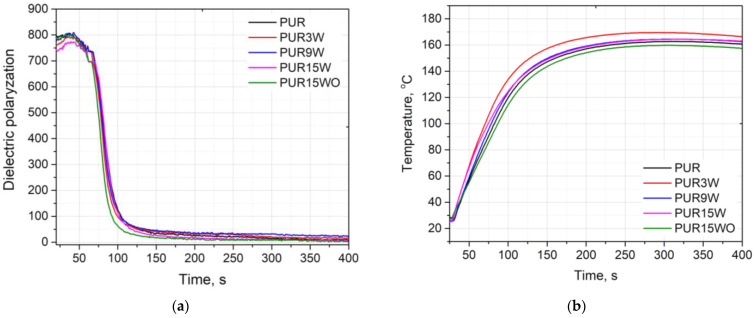
Influence of vermiculite on dielectric polarization (**a**) and temperature (**b**).

A modification of a PUR system with fillers can have an influence on the reactivity of the system. Changes in the reactivity of the PUR system were analyzed using the FOAMAT device. The reactivity of the PUR system is illustrated by the changes in the dielectric polarization curve. The systems with higher reactivity are characterized by a faster dielectric polarization reduction. The changes in dielectric polarization, as well as the temperature of the reaction mixture during the foaming process, are shown in Figure 5.

The results indicate that a modification of the reference system with vermiculite, regardless of its content, did not significantly affect the reactivity of the PUR system, which was confirmed by observations of the dielectric polarization and temperature changes. A similar effect was observed in our earlier work, where the PUR foams were modified with thermoset polyester composite waste [24]. A slight influence on the foaming process is extremely important as the foaming process determines the cell structure of the foam and its subsequent functional properties. In the literature, an effect of decreased reactivity of the PUR foam modified with a waste filler has been described. Formela et al. [25] applied the brewer’s spent grain and ground tire rubber in rigid PUR foams, causing modification, which resulted in a decrease in the reaction rate. The rise time and tack-free time of the rigid PUR foams modified with 20 wt.% of the brewer’s spent grain were two and almost three times longer than those of the unmodified system, respectively.

### 3.4. Properties of Rigid PUR Foams Modified with Vermiculite

The cellular structure of porous materials has a significant effect on their properties and depends on many factors such as: premix viscosity, their modification by fillers, method of foaming etc. [26].

**Figure 6:**
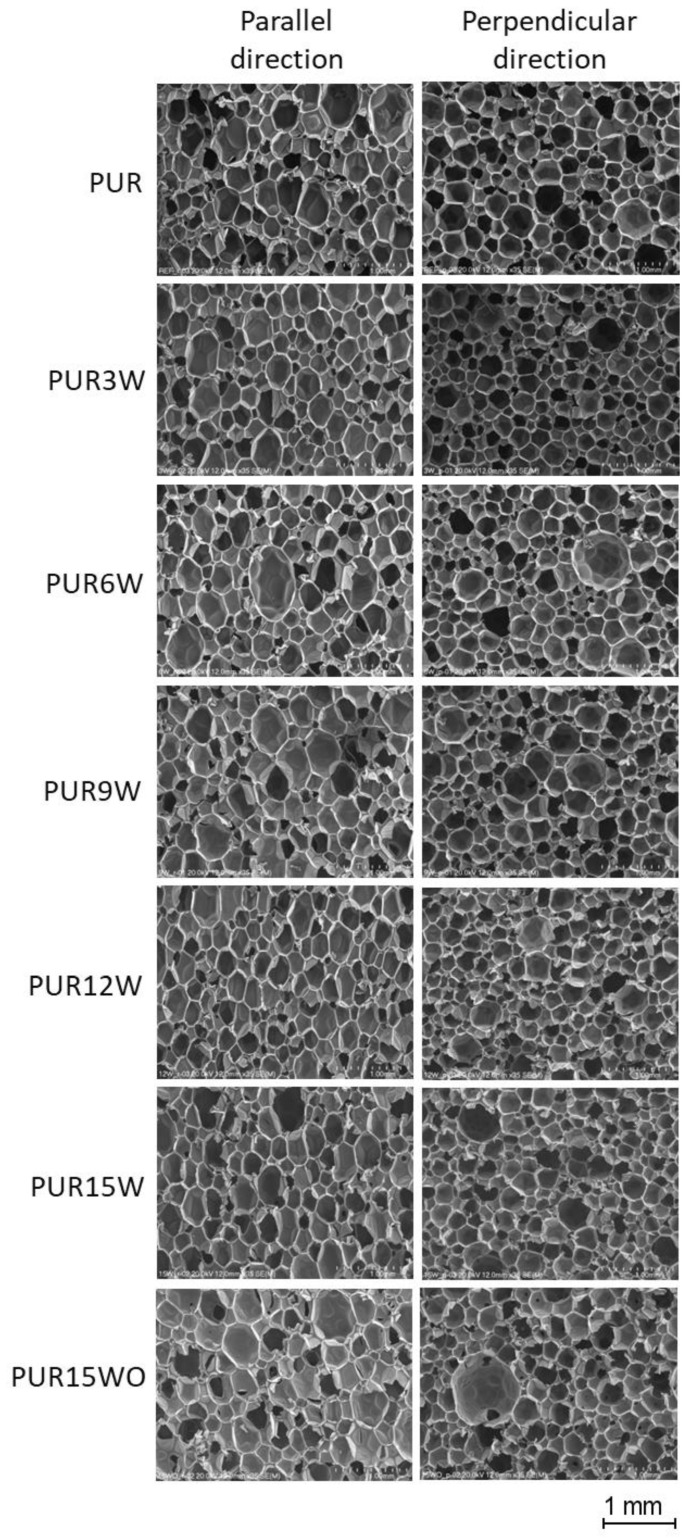
The cellular structure of PUR foams and foams modified with vermiculite.

**Figure 7:**
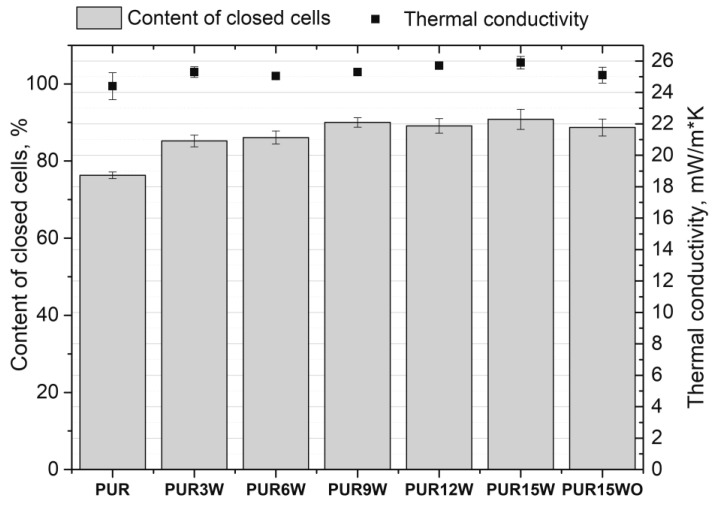
Content of closed cells and thermal conductivity of foams modified with vermiculite.

All the tested foams exhibited well-developed hexagonal cell structures (Figure 6). The modification of the reference system (PUR) with the vermiculite and modified vermiculite improved the morphology of the PUR composites, generally reducing cells’ average diameters (Table 3). However, the changes are insignificant and within the measurement error. The contents of closed cells were higher for the foams modified with vermiculite (Figure 7). The content of closed cells is important from the thermal insulation properties point of view. Closed-cell foams are characterized by lower values of the thermal conductivity coefficient compared to open-cell foams.

Based on the results presented in Figure 7, it can be observed that the content of closed cells increased from 76% for the reference material (PUR) to 91% for the foams with the highest vermiculite content (PUR15W). However, the value of the thermal conductivity coefficient is characterized by the highest value for a given material (PUR15W) despite the highest closed cell content. Such an effect may be related to the highest apparent density and relatively high AI of PUR15W material. It was observed that the material into which the WO was introduced has a higher apparent density, while the thermal conductivity of this material is lower than that of the material containing the same amount of W. This can be explained by the lowest AI, which means this PUR15WO foam has a less anisotropic structure. This structure limits heat transport through the foamed material. Depending on the type of filler, the effect on the variation of the foam’s apparent density may be different. Natural fillers, such as flax and hemp fibers, can decrease the apparent density of foams as a result of the moisture present in them (carbon dioxide is generated in the reaction of water and isocyanate). In the case of fillers characterized by high density, e.g., carbon fibers, montmorillonite, or other inorganic fillers, the apparent density of PUR foams is increased [27].

**Figure 8:**
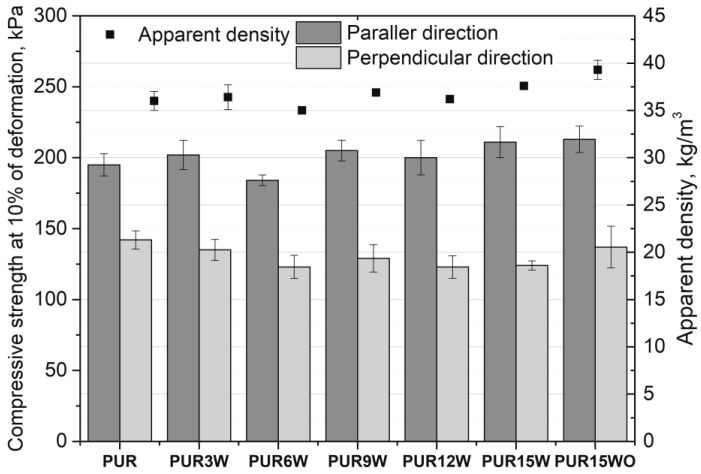
Apparent density and compressive strength of foams modified with vermiculite.

The PUR foams modified with vermiculite were characterized by an apparent density (Figure 8) in the range of 35–39 kg/m^3^. The foams with modified vermiculite (PUR15WO) had the highest apparent density. However, differences among tested foams are not significant, taking into account the standard deviation. The compressive strength (Figure 8) measured in the direction parallel to the direction of the foam growth is characterized by greater values than when measured in the perpendicular direction. These differences are due to the anisotropic nature of the cellular structure of the PUR foams obtained. The compressive strength results are comparable with those obtained for the reference material. There is a slight increase in the mechanical strength of the foams with the highest vermiculite content. This effect can be related to a slight increase in the apparent density of the materials with the highest filler content.

**Figure 9:**
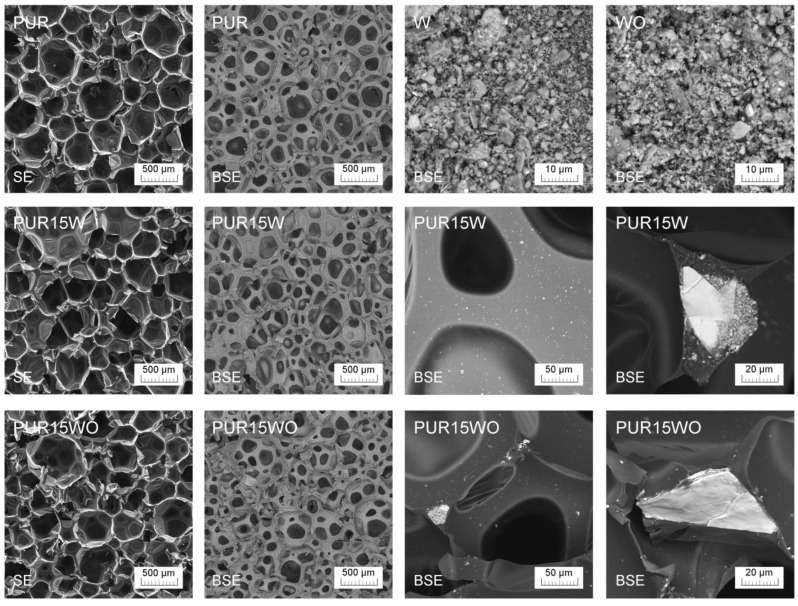
SEM images made for reference PUR and PUR 15W and PUR 15WO in SE and BSE mode showing the dispersion of the filler in a foamed polymeric matrix.

Figure 9 shows the collectively presented SEM images made for the reference sample (PUR), fillers (W, WO), and composites with the highest concentration of fillers (PUR15W, PUR15WO). The analysis was performed with SE and BSE modes to increase the visibility of the filler particles in the matrix. It can be concluded that the filler is well distributed in the polymer matrix. There are no torn-out inorganic fractions that could result from improper adhesion. Moreover, it should be emphasized that there are no agglomerated structures. Filler particles smaller than 1 µm are evenly distributed, while larger particles are embedded in the walls of the foam cells, especially in the nodes. Therefore, their localization does not weaken the cell structure, and no voids were noted in the interphase area, which could suggest a lack of adhesion between a polymer and a filler.

The aesthetics of the final products often plays an important role in selecting materials by design teams. The analysis of color, which is one of the primary criteria for the qualitative assessment of products, is essential from the point of view of the potential of selected product groups [28,29]. Table 4 summarizes the L*, a*, and b* chromatic parameters, describing the color in the CIELab space of the produced foams with different vermiculite contents. Additionally, the results of the total color change were calculated according to Equation (1). Even the smallest addition of the filler caused significant color changes, taking into account the criteria described in the ISO 2813 standard and the literature [29]. Based on the low values of standard deviations, all the foams were characterized by lower luminescence and had a brown shade with a uniform color. This also confirms the good compatibility and miscibility of the PUR-W/WO. It should be emphasized that in the case of the foams with the highest filler concentration, no significant changes in ΔE between the batches made with expanded and H_2_O_2_ treated with vermiculites were noted.

**Figure 10:**
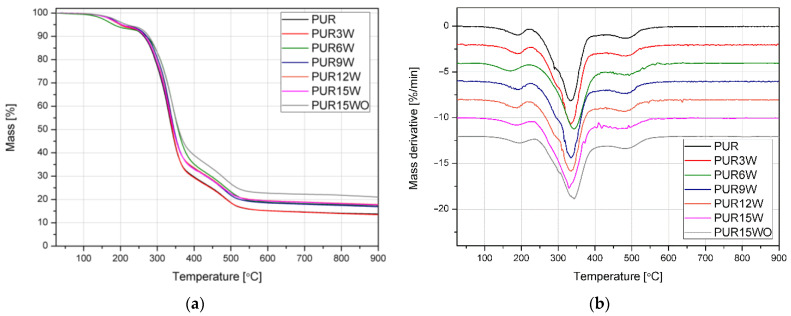
TG (**a**) and DTG (**b**) curves of polyurethane samples with various contents of vermiculite.

The results of the thermogravimetric analysis are presented in the form of TG, and DTG graphs in Figure 10. Table 5 collectively shows thermal parameters, such as temperature at 5%, 10%, 25%, and 50% mass loss, residual mass at 900 °C, and data describing peaks observed at the first derivative of the group. The courses of the TG and DTG curves (Figure 10) indicate the three-step course of the thermal degradation process of the rigid PUR foams. The first stage of degradation in the temperature range from 110 to 220 °C, with the maximum between 170 and 190 °C, is related to the evaporation of residual water and low molecular weight products in the PUR foam [30]. The observed dominant degradation stage with the maximum process intensity observed around 320 °C is associated with hard-segment decompositions. As demonstrated by Jiao et al. [30], in a narrow range between 320 °C and 350 °C, isocyanate monomers almost disappear. First, N-H bonds are degraded, resulting in the degradation of hard segments, then C-H bonds from methyl and methylene groups. The last step of decomposition observed in the temperature range of 370–420 °C corresponds to the degradation of ester bonds in polyols [31,32]. It should be emphasized that the introduction of the filler caused shifts in decomposition stages; however, it did not affect its mechanism, which proves that there were no significant changes in the chemical structure of the PUR composition. Based on the conducted research, it can be clearly stated that adding unmodified and hydrogen peroxide-modified vermiculite improved the thermal properties of the composites as compared to the unmodified PUR foam (enhanced T_5%_ and yield of residue). In the case of introducing expanded vermiculite into the PUR matrix, it is difficult to find a clear trend related to the amount of the filler and only an apparent effect of increasing thermal stability, especially distinct as evaluated at a residue (Table 5). On the other hand, using a two-step treatment based on thermal development and subsequent immersion in concentrated peroxide significantly improved thermal stability of PUR-based composite. This may be related to the improvement in the filler dispersion resulting from its structure modification described in the earlier paragraph, with the formation of sheets of reduced size.

**Figure 11:**
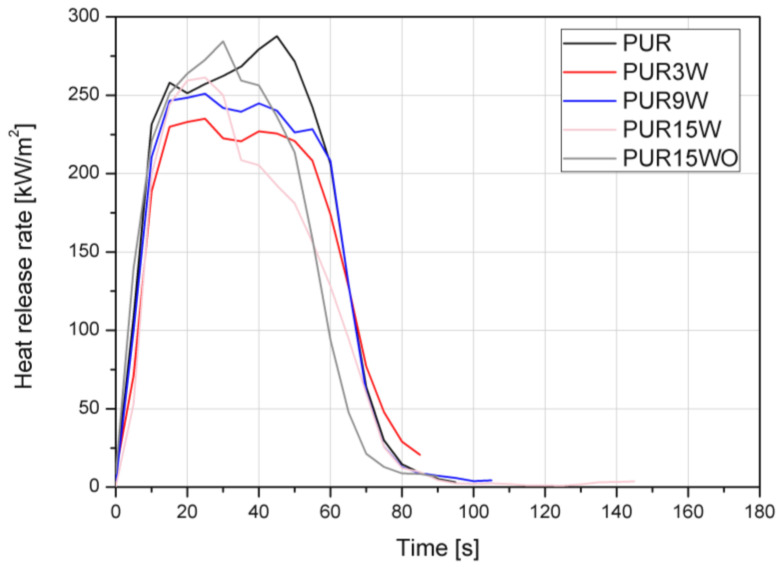
Representative heat release rate curves of PU foams modified with vermiculite.

The cone calorimeter test is a small-scale test employed to observe a comprehensive set of fire features in a well-defined fire scenario [33]. The measurement provides the value of parameters, such as time to ignition (TTI), heat release rate (HRR), the maximum average rate of heat emission (MARHE), total heat release (THR), effective heat of combustion (EHC), specific extinction area (SEA), and total smoke release (TSR). The HRR curves for the materials investigated in this study are illustrated in Figure 11, while detailed data are summarized in Table 6.

The HRR curves suggest that all PUR foams ignited at a comparable time, which was confirmed by the time to ignition values presented in Table 6. Their cellular structure and low thermal conductivity strongly influence the burning behavior, and TTI reached only 5 ± 1 s. The heat release rate is an essential parameter to estimate fire development, intensity, and spreading. The trend of the HRR curve demonstrates the burning behavior of the materials as a function of time. The curve of the PUR exhibits quite a broad peak with a maximum average value of 288 kW/m^2^. It can be observed that vermiculite led to a change in the curves trend from characteristic for thick non-charring to thick charring ones [33]. The lowest pHRR of 237 kW/m^2^ (reduction of 18%) was obtained for the composites PUR3W, so the values were independent of the filler content and its modification. MARHE, as an indicator determined from HRR, is used to estimate the hazard of developing fires. Consequently, lower MARHE was obtained for samples with lower pHRR. Vermiculite is a filler known for its flame-retardant effects [34,35,36]; however, no change in LOI values as a result of W or WO addition was observed.

**Figure 12:**
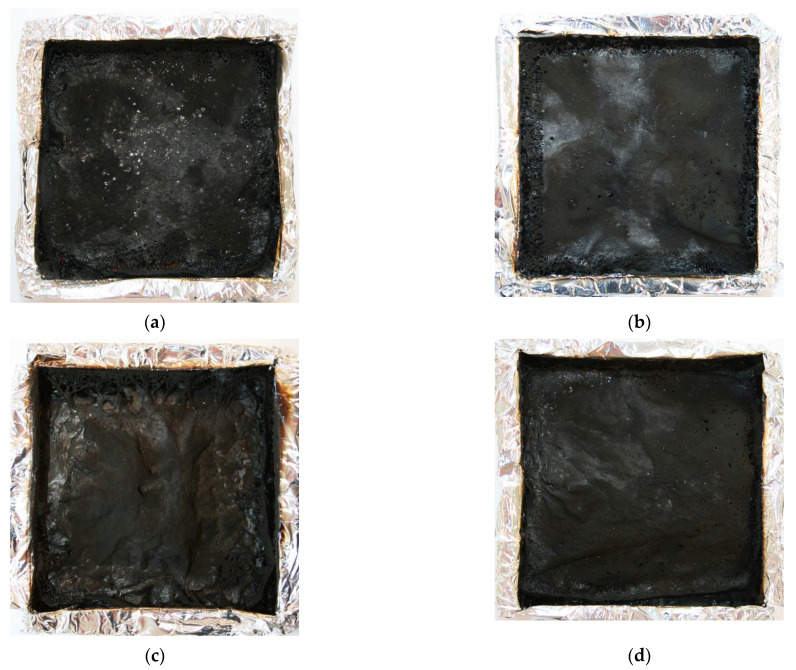

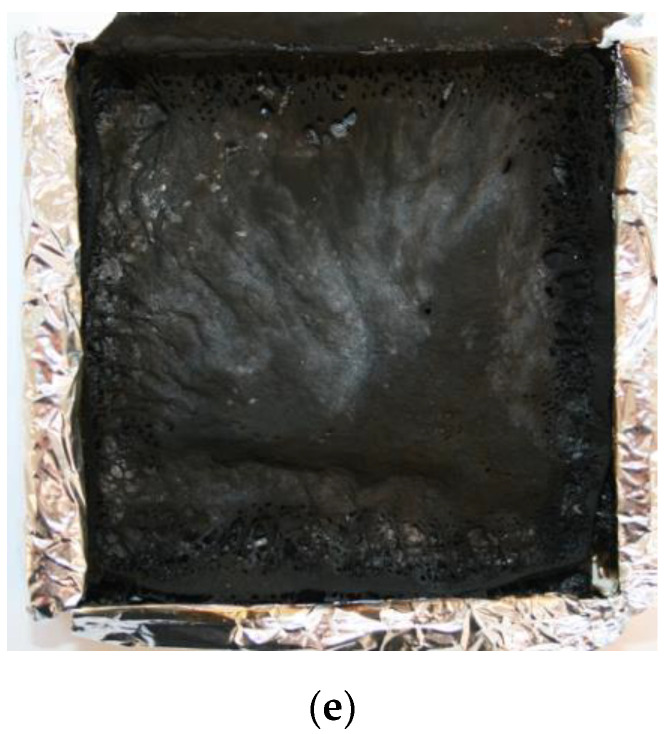
Photographs of samples after cone calorimetry tests (**a**) PUR, (**b**) PUR3W, (**c**) PUR9W, (**d**) PUR15W, (**e**) PUR15WO.

The integral of HRR over time expresses the total heat output, i.e., the THR [33]. The vermiculite addition caused a non-linear decrease in THR, suggesting incomplete combustion affected by char formation or reduced combustion efficiency [37]. Since there was no change in EHC, as well as according to the increased yield of residue (Table 6, Figure 10a), action probably occurred in the condensed phase. Moreover, the content of triethyl phosphate, which is a phosphorus flame retardant active mainly in the gas phase, was the same for all materials. The analysis of the photographs confirms that the presence of vermiculite facilitated the formation of a more compact structure, and the number of holes decreased with an increase in the amount of vermiculite (Figure 12). Similar to the carbonaceous char, inert residue from inorganic fillers works as a barrier and additionally replaces polymer, reducing the fuel release [30]. Probably, the residues of the investigated materials were the origin of both effects. 

During a fire, smoke is of great importance as it reduces visibility and makes an escape more challenging [38,39,40]. Considering the SEA values together with the standard deviation, it can be concluded that the use of vermiculite did not change this parameter. The lowest SEA, which corresponds to the surface light-absorbing particles of smoke [39], was recorded for composites PUR3W and amounted to 787 m^2^/kg. In turn, the TSR of all composites was reduced compared to the unmodified foam, and the highest decrease reached approximately 13% (PUR15W). Presumably, this is due to the increased amount of the material remaining in the condensed phase.

## 4. Conclusions

Rigid polyurethane foams modified with the thermally expanded vermiculite were successfully obtained. The apparent density of foams was comparable in the range of 35–39 kg/m^3^. The impact of different filler contents on the foaming process, cellular structures, physical-mechanical properties, thermal stability, and flammability of porous composites was determined. It was found that a modification of the reference system with thermally expanded vermiculite did not significantly affect the reactivity of the polyurethane system, which was confirmed by similar trends observed for dielectric polarization changes. A slight increase in reactivity was observed, confirmed by a faster decrease in dielectric polarization for the polyurethane system with modified vermiculite by soaking in H_2_O_2_. The modification of the reference system with vermiculite and modified vermiculite improved the morphology of the porous polyurethane composites and increased the content of closed cells.

The coefficient of thermal conductivity of reference foam and foams modified with vermiculite was in the range 24–26 mW/mK, which makes such materials interesting for heat-insulating applications. The use of vermiculite up to 15 wt.% did not significantly influence other tested properties (thermal, mechanical, fire) of the foams but could make such modified materials cheaper and useful for industrial application.

## Figures and Tables

**Table 1:** The formulation of the reference polyurethane foam.

Component	Mass, g
Rokopol RF-551	100
PMDI	181
L6633	1.5
Polycat 9	2
Water	4
TEP	20
Vermiculite	3, 6, 9, 12, 15% of the mass of the polyol

**Table 2:** Characteristic of vermiculite grades based on BET analysis.

Material	S_BET_, m^2^/g	S_EXT_, m^2^/g	S_MIC_, m^2^/g	4V/A, nm	V_P_, cm^3^/g	V_MIC_, cm^3^/g
W	12.58	12.77	0.81	14.29	0.048556	0.000257
WO	17.09	16.56	0.53	12.64	0.054028	0.000072

**Table 3:** Influence of vermiculite on the diameter of cells in PUR foams.

Symbol	Parallel Direction, µm	Perpendicular Direction, µm	AI *
PUR	309.4 ± 105.6	281.3 ± 89.2	1.10
PUR3W	287.4 ± 112.4	248.1 ± 83.9	1.16
PUR6W	303.8 ± 122.4	254.2 ± 94.5	1.19
PUR9W	317.7 ± 119.3	273.7 ± 95.9	1.16
PUR12W	285.7 ± 105.6	260.9 ± 92.4	1.10
PUR15W	299.2 ± 107.1	264.6 ± 91.3	1.13
PUR15WO	291.1 ± 104.4	277.5 ± 103.6	1.05

AI *—anisotropy index calculated as a ratio of cell diameters measured in cross-sections parallel and perpendicular to the foam rise directions.

**Table 4:** CIELab color parameters and ΔE of PUR and PUR-W/WO composites.

Sample	L*	a*	b*	ΔE
PUR	85.62 ± 0.93	−3.05 ± 0.39	13.94 ± 0.85	-
PUR3W	79.08 ± 1.13	1.24 ± 0.16	14.44 ± 0.64	7.86
PUR6W	74.92 ± 1.74	3.29 ± 0.26	14.30 ± 0.64	12.46
PUR9W	70.97 ± 1.56	4.48 ± 0.34	14.89 ± 0.59	16.61
PUR12W	69.89 ± 1.06	5.42 ± 0.32	14.27 ± 0.50	17.89
PUR15W	65.61 ± 1.51	6.66 ± 0.40	15.03 ± 0.59	22.29
PUR15WO	65.46 ± 1.25	7.05 ± 0.36	16.29 ± 0.61	22.69

**Table 5:** Thermal parameters obtained by TGA for PUR and PUR-based composites modified with various vermiculite contents.

Sample	T5%, °C	T10%, °C	T25%, °C	T50%, °C	Residue at 900 °C, %	DTG 1st Peak, %/min; °C	DTG 2nd Peak, %/min; °C	DTG 3rd Peak, %/min; °C
PUR	198.7	263.8	304.2	339.7	13.76	−0.98; 190.5	−8.16; 332.2	−1.33; 481.6
PUR3W	199.8	266.7	307.6	341.0	13.49	−1.03; 190.0	−8.73; 333.5	−1.27; 472.7
PUR6W	181.7	270.9	319.0	356.7	17.38	−0.88; 170.2	−7.23; 343.0	−1.40; 493.0
PUR9W	205.1	271.4	312.4	345.5	16.91	−0.94; 190.2	−8.39; 335.1	−1.38; 486.8
PUR12W	199.7	266.8	308.6	344.1	17.86	−0.94; 188.2	−7.83; 335	−1.31; 481.9
PUR15W	204.8	266.6	307.1	343.8	17.61	−0.81; 187.4	−7.70; 329.2	−1.24; 460;8
PUR15WO	217.8	277.1	318.5	358.4	21.06	−0.75; 194.6	−6.88; 343.0	−1.37; 481.6

**Table 6:** Cone calorimeter test results of the tested foams.

Sample	TTI,	pHRR,	MARHE,	THR,	EHC,	SEA,	TSR,	Residue,	LOI,
	s	kW/m^2^	kW/m^2^	MJ/m^2^	MJ/kg	m^2^/kg	m^2^/m^2^	%	%
PUR	4 ±1	288 ± 19	233 ± 11	15 ± 1	16 ± 0	811 ± 22	768 ± 61	15.2 ± 2	21.5 ± 0.1
PUR3W	6 ± 3	237 ± 42	188 ± 34	13 ± 1	16 ± 0	787 ± 55	666 ± 78	13.3 ± 1	21.5 ± 0.1
PUR9W	4 ± 1	284 ± 19	222 ± 5	14 ± 0	16 ± 0	822 ± 25	707 ± 32	16.5 ± 3	21.5 ± 0.1
PUR15W	5 ± 1	266 ± 6	207 ± 11	13 ± 1	16 ± 0	824 ± 14	668 ± 39	21.4 ± 2	21.5 ± 0.1
PUR15WO	4 ± 1	274 ± 27	222 ± 24	14 ± 0	16 ± 0	810 ± 30	699 ± 10	19.6 ± 0	21.5 ± 0.1

## Data Availability

The data presented in this study are available on request from the corresponding author.
